# Seasonal and Meteorological Drivers of Hand, Foot, and Mouth Disease Outbreaks Using Data-Driven Machine Learning Models

**DOI:** 10.3390/tropicalmed10020048

**Published:** 2025-02-06

**Authors:** Pakorn Lonlab, Suparinthon Anupong, Chalita Jainonthee, Sudarat Chadsuthi

**Affiliations:** 1 Department of Physics, Faculty of Science, Naresuan University, Phitsanulok 65000, Thailand; pakornl64@nu.ac.th; 2 Department of Chemistry, Mahidol Wittayanusorn School (MWIT), Salaya, Nakhon Pathom 73170, Thailand; suparinthon.anu@mwit.ac.th; 3 Research Center for Veterinary Biosciences and Veterinary Public Health, Faculty of Veterinary Medicine, Chiang Mai University, Chiang Mai 50100, Thailand; chalita.j@cmu.ac.th

**Keywords:** hand, foot, and mouth disease, outbreak detection, meteorological factors, machine learning

## Abstract

Hand, Foot, and Mouth Disease (HFMD) predominantly affects children under the age of five and remains a significant public health concern in the Asia-Pacific region. HFMD outbreaks are closely linked to seasonal changes and meteorological factors, particularly in tropical and subtropical areas. In Thailand, a total of 657,570 HFMD cases were reported between 2011 and 2022 (12 years). This study aimed to identify the high- and low-risk HFMD outbreak areas using machine learning models: Logistic Regression (LR), Support Vector Machine (SVM), Decision Tree (DT), Random Forests (RF), Gradient Boosting Machine (GBM), and Extreme Gradient Boosting (XGBoost). Our findings showed that the XGBoost model outperformed the other models in predicting unseen data and defining the best model. The best model can be used to detect high-risk outbreak areas and to explore the relationship between meteorological factors and HFMD outbreaks. The results highlighted the seasonal distribution of high-risk HFMD outbreak months across different provinces in Thailand, with average maximum temperature, average rainfall, and average vapor pressure identified as the most influential factors. Furthermore, the best model was used to analyze HFMD outbreaks during the COVID-19 pandemic, showing a notable reduction in high-risk outbreak months and areas, likely due to the control measures implemented during this period. Overall, our model shows great potential as a tool for warnings, providing useful insights to help public health officials reduce the impact of HFMD outbreaks.

## 1. Introduction

Hand, Foot, and Mouth Disease (HFMD) is a contagious viral infection that occurs worldwide, primarily caused by Coxsackievirus and Enterovirus A71, mainly affecting children under five. In the Asia-Pacific region, HFMD outbreaks have been reported as a severe public health concern affecting countries such as China [1], Taiwan [2], Malaysia [3], Vietnam [4], Singapore [5], and Thailand [6]. Clinically, HFMD is characterized by fever, oral ulcers, and rashes on the hands, feet, and buttocks [7]. In the absence of specific antiviral treatments, the management of HFMD primarily emphasizes symptomatic relief. Prompt identification and isolation of infected individuals are essential for mitigating the transmission of the disease.

HFMD outbreaks have been closely linked to seasonal changes and meteorological factors, particularly in tropical and subtropical regions [8]. Several studies have demonstrated the influence of temperature, humidity, and rainfall on HFMD incidence [8]. For instance, research in China has shown that higher temperatures and elevated humidity levels are significantly associated with increased HFMD cases [9,10]. Similarly, a study by Xu and co-authors in Japan identified temperature as a key driver of HFMD transmission, with peak incidences observed during summer [11]. In Vietnam, increased temperatures and rainfall have also been linked to a higher incidence of HFMD cases [12].

In Thailand, Phromsin et al. [13] demonstrated that temperature and humidity were key climatic factors influencing HFMD outbreaks in Northern Thailand, where rainfall and humidity were most important in the Central region. It also highlighted the potential role of topography in disease distribution and recommended applying these findings for outbreak control and epidemic prediction. A study conducted in Northern Thailand found that HFMD outbreaks tended to occur roughly every two years during the rainy and cold seasons [14]. This suggests a strong correlation between HFMD incidence and climatic factors.

Recently, machine learning (ML) techniques have been used to analyze large epidemiological datasets and predict disease outbreaks. ML approaches have gained increasing attention for developing models that offer improved predictive power [15]. Classification algorithms such as Logistic regression (LR), Support Vector Machine (SVM), Decision tree (DT), Random Forest (RF), Gradient Boosting Machine (GBM), and Extreme Gradient Boosting (XGBoost) have been widely utilized to classify data based on features such as geographic location, population density, and climate factors [16]. For example, in Malaysia, an SVM with a linear kernel was the best model for predicting dengue outbreaks based on climate variables, outperforming the Classification and Regression Tree (CART) [17]. Another study in Malaysia employed Bayesian networks for dengue outbreak prediction [18]. In Thailand, ML techniques were applied to predict dengue vector abundance [19]. Moreover, models such as Backward Propagation Neural Network (BPNN), RF, and GBM have been developed to map the probability of Zika epidemic outbreaks [20]. XGBoost proved to be a robust model for predicting West Nile virus transmission in Europe from 2010 to 2019 [21]. For HFMD outbreak prediction, ridge regression, K-Nearest Neighbors (KNN), RF, and Recurrent Neural Networks (RNN) have been applied using morbidity data in China [22]. LR was also used to analyze the association between various environmental factors and the high and low occurrence rates of HFMD [23]. Despite extensive research on the seasonal dynamics of HFMD, the application of ML methods for outbreak prediction has received limited attention. Moreover, studies focusing on the utilization of weather variables for detecting HFMD outbreaks across Thailand remain underexplored.

This study aimed to address the gap in HFMD outbreak prediction by comparing the performance of six machine learning classifiers: LR, SVM, DT, RF, GBM, and XGBoost. Our main objective was to identify the best model for accurately classifying high-risk outbreak areas, helping raise public health awareness, and supporting timely preventive measures during periods of increased risk. Furthermore, the best model will be used to investigate the relationship between meteorological variables and HFMD outbreaks in Thailand. The study also aimed to assess predictive performance during and after the COVID-19 pandemic (2019–2022), a period that significantly influenced HFMD transmission dynamics. The proposed model holds the potential to be integrated into public health decision-making frameworks, offering valuable insights to mitigate the impact of HFMD outbreaks and guiding resource allocation and targeted interventions.

## 2. Materials and Methods

### 2.1. Data Collection

In this study, monthly reported cases of HFMD at the provincial level from 2011 to 2022 were obtained from the National Disease Surveillance (report 506) database, maintained by the Bureau of Epidemiology, Department of Disease Control, Ministry of Public Health, Nonthaburi, Thailand [24]. The reported cases were collected from the passive surveillance system based on established national reporting criteria. Monthly population data for each province were retrieved from the Office of Registration Administration, Department of Provincial Administration [25]. Population data for Bueng Kan province (one of the 77 provinces) were unavailable for January and February 2011. To address this issue, data for these two months were excluded from the analysis.

Meteorological data, including monthly minimum and maximum temperatures, rainfall, soil moisture, vapor pressure, and wind speed, were extracted from the TerraClimate database (https://www.climatologylab.org/terraclimate.html, accessed on 9 January 2024) [26], which provides data at a spatial resolution of approximately 4 km (1/24th degree). The meteorological data for each province were calculated as the average or summation of the values from the grids located within the province’s boundaries. Descriptions of the meteorological variables are provided in Table 1.

### 2.2. Data Processing and Creating the HFMD Outbreak Variable

In this work, feature data were pre-processed by applying standardization to scale the variables. After standardization, each variable has a mean of zero and a standard deviation of one. This transformation stabilizes the variance and makes the data more suitable for modeling [27]. These pre-processing steps facilitated robust model validation across various time periods and provinces, ensuring a comprehensive assessment of model performance and generalizability.

To create the HFMD outbreak classification, the target variable (HFMD incidence rate) was assigned a value of 1 for a high-risk outbreak and 0 for a low-risk outbreak. The epidemic threshold for each province was defined using the median incidence rate per 100,000 population to account for variations in baseline incidence across provinces and ensure a balanced classification of high- and low-risk months. The median was chosen because it is robust to outliers and prevents skewing caused by extreme values, unlike the mean or higher percentiles. A month was classified as a high-risk outbreak if its incidence rate exceeded the median value for that province. This binary classification approach was essential for predicting outbreak risk across different provinces and time periods.

### 2.3. Model Selection

**Figure 1:**
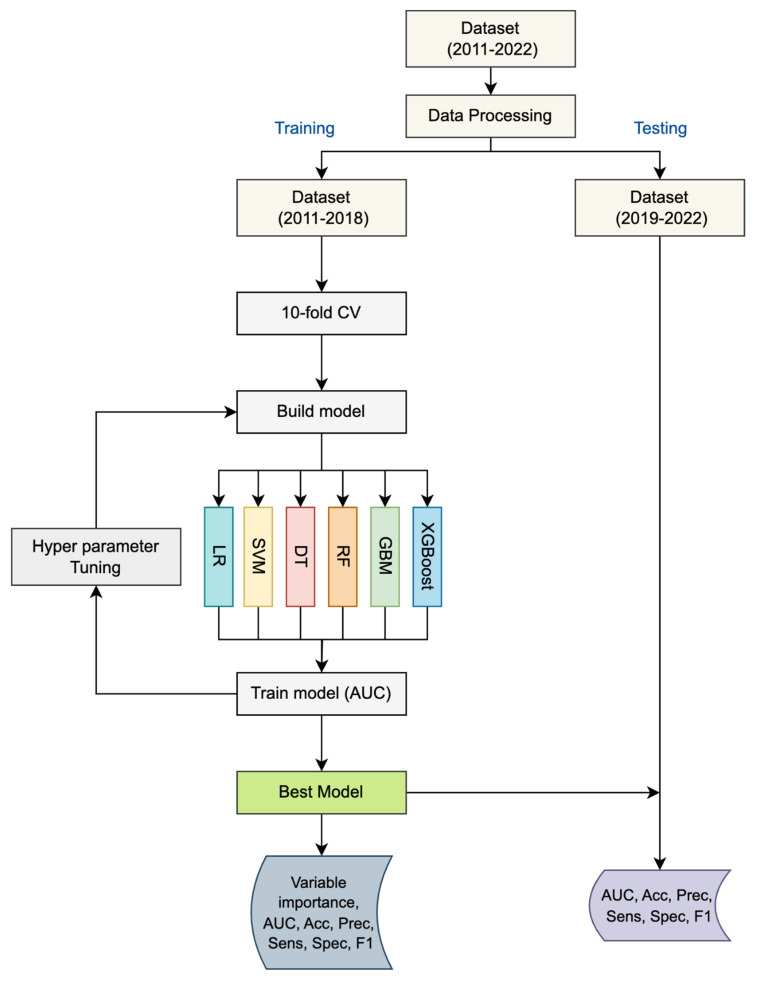
The ML framework for HFMD outbreak detection. Six models used Logistic Regression (LR), Support Vector Machine (SVM), Decision Tree (DT), Random Forests (RF), Gradient Boosting Machine (GBM), and Extreme Gradient Boosting (XGBoost).

To select the best model, the data were divided into two subsets: data from 2011 to 2018 were used for training, while data from 2019 to 2022 were used for testing and analyzing the impact of the COVID-19 pandemic. Six machine learning classifiers were evaluated: LR, SVM, DT, RF, GBM, and XGBoost. Each model was evaluated using multiple performance metrics to identify the best model for predicting HFMD outbreak risks during 2019–2022. The overall model framework is illustrated in Figure 1.

Each model was trained and validated using 10-fold cross-validation (CV) to minimize bias and variance. The data from 2011 to 2018 were randomly partitioned into 10 equal subsets, with the model trained on nine subsets and validated on the remaining one. This process was repeated across all folds to ensure robust evaluation. Hyperparameters were tuned using grid search, and the optimal configuration was selected based on the highest Area Under the Receiver Operating Characteristic (ROC) Curve (AUC) score. Models with an AUC greater than 0.8 were considered good fits [19] and were subsequently used for predictions on the test dataset. Details of the models and their hyperparameters are summarized in Table S1.

LR is a statistical model that classifies binary outcomes (Y = 1 for a high-risk outbreak or 0 for a low-risk outbreak) using the logit link function to transform the linear predictor into a probability [28].

SVM aims to find an optimal hyperplane that separates classes with the maximum margin [29]. In this study, an SVM with a radial basis function (RBF) kernel was used due to its effectiveness in handling non-linear decision boundaries.

DT uses a tree structure to classify data based on feature splits that minimize impurity, measured by the Gini index. The algorithm selects the feature and split point that results in the greatest impurity reduction.

RF, GBM, and XGBoost are ensemble learning methods widely used for classification tasks. Ensemble methods combine predictions from multiple learners to improve accuracy and reduce overfitting.

RF constructs multiple decision trees during training using bootstrap aggregation (bagging), where each tree is trained on a randomly sampled subset of the data (with replacement). Additionally, at each split within a tree, RF selects a random subset of features, introducing diversity among the trees. Predictions are aggregated through majority voting for classification or averaging for regression, making RF robust and accurate. This method was developed by Leo Breiman [30].

GBM builds an ensemble of weak learners sequentially, with each new learner trained to correct the residual errors of the previous ones. This iterative process minimizes a specified loss function, such as the logistic loss function for binary classification, to reduce classification errors through gradient descent [31,32]. While GBM improves predictive accuracy, it is prone to overfitting.

XGBoost is an optimized implementation of the gradient boosting framework that is designed for high performance and efficiency. Developed by Chen and Guestrin [33], XGBoost offers additional hyperparameters for tuning, making it suitable for complex datasets and large-scale problems [21].

The hyperparameter tuning process continued until the specified stopping criterion was met. The final model, selected based on the highest AUC score during validation, was used to predict the test dataset.

### 2.4. Evaluation Metrics

To evaluate the performance of the model for HFMD outbreak detection, we used five metrics: accuracy (Acc), precision (Prec), sensitivity/recall (Sens), specificity (Spec), and F1 score (F1). First, Acc represents the proportion of correct predictions over the total number of predictions. Second, Prec refers to the proportion of correctly predicted high-risk outbreak months out of all months predicted as high-risk outbreaks. Sens is the proportion of actual high-risk outbreak months that were correctly predicted as high-risk outbreaks. Spec is the proportion of actual low-risk outbreak months that were correctly predicted as low-risk outbreaks. Finally, F1 is the harmonic mean of precision and recall. The set of metric equations is defined as follows:

(1) $$Accuracy=\frac{TP+TN}{TP+FP+FN+TN},\;Precision=\frac{TP}{TP+FP},\;Sensitivity/Recall=\frac{TP}{TP+FN},\;Specificity=\frac{TN}{TN+FP},\;F1\;Score=\frac{2\times Precision\times Recall}{Precision+Recall},$$

where *TP*, *TN*, *FP*, and *FN* are True Positive, True Negative, False Positive, and False Negative, respectively.

### 2.5. Softwares

We used R program version 4.4.1 [34] with package caret 6.0–94 [35], rpart 4.1.19 [36], and gbm 2.2.2 [37]. We used pROC 1.18.5 [38] to evaluate the performance metrics and determine variable importance ranks. The package tidyverse 2.0.0 [39], ggpubr 0.6.0 [40], spdep 1.2–8 [41], corrplot 0.92 [42] and sf 1.0–13 [43] used for data analysis and visualization.

## 3. Results

### 3.1. Descriptive Data

Table 2 presents a descriptive analysis of the HFMD incidence rates and meteorological data outlined in Table 1. Between 2011 and 2022 (12 years), a total of 657,570 HFMD cases were reported in Thailand, with the highest incidence rate recorded at 179.78 cases per 100,000 people located in Surin Province in August 2022. The monthly average minimum temperature across all provinces was 22.34 °C, ranging from 10.51 °C to 27.31 °C, while the monthly average maximum temperature was 32.16 °C, with a range of 24.13 °C to 40.00 °C. The monthly averages for precipitation, soil moisture, vapor pressure, and wind speed in Thailand were 129.52 mm, 161.42 mm, 2.66 kPa, and 1.36 m/s, respectively. These variables exhibited large standard deviations, reflecting the significant climatic diversity across the country. Figures S1–S10 present heatmaps illustrating the HFMD incidence rates and the corresponding meteorological data for each province and month.

**Figure 2:**
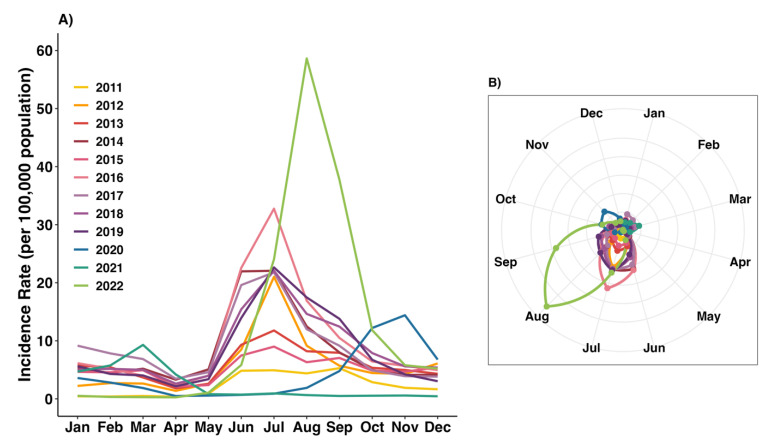
Seasonal (**A**) line plot and (**B**) Polar plot for monthly HFMD incidence rates averaged across all provinces in Thailand during 2011–2022.

Figure 2 illustrates the seasonal pattern of HFMD incidence rates observed annually throughout the study period. Elevated incidence rates were consistently recorded during the wet season, from June to September, between 2011 and 2019 (Figure 2A). The highest monthly peak occurred in July 2016, reaching 31.06 cases per 100,000 people. During the COVID-19 pandemic, HFMD incidence rates significantly declined, with zero cases reported in some months of 2020. However, the disease reemerged between September and December of the same year. In 2021, the incidence rates remained low, nearly reaching zero. Following the end of the COVID-19 outbreak in 2022, HFMD incidence rates increased sharply, peaking in August at 54.12 cases per 100,000 people—a notable difference in seasonal pattern from previous years (Figure 2B).

**Figure 3:**
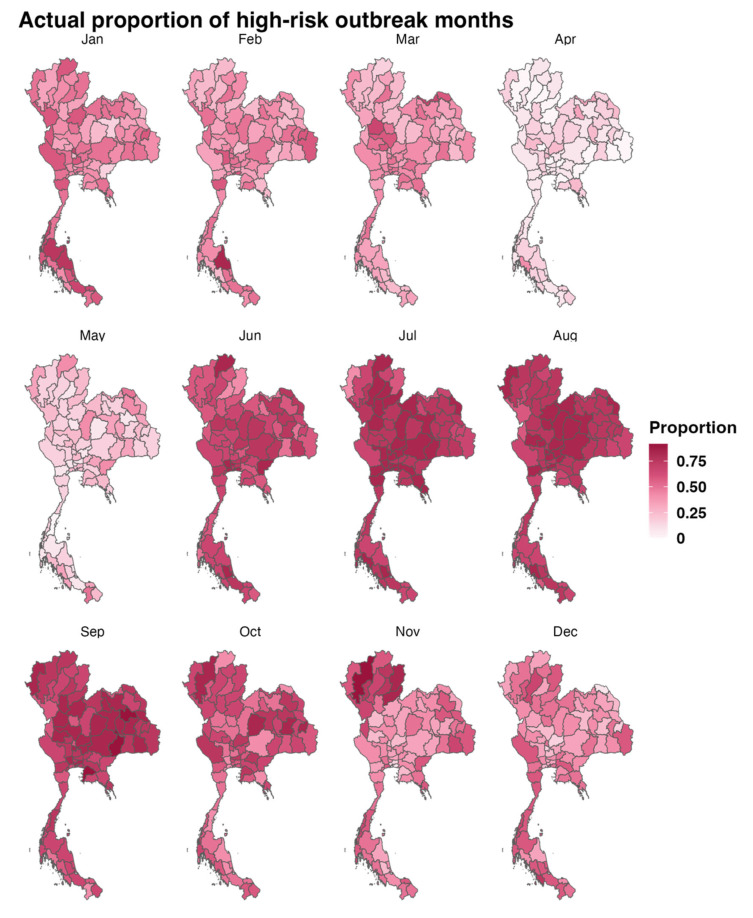
Maps of the proportion of high-risk HFMD outbreaks each month across 12 years (2011–2022), showing the seasonality and the distribution of the HFMD epidemics. The intensity of the pink color represented the proportion of high-risk HFMD outbreaks.

The proportion of high-risk HFMD outbreak months was calculated as the number of outbreaks in a given month divided by the total number of studied years (12 years). For example, in January in Bangkok, if there were six high-risk outbreak months for 12 years, the proportion of HFMD outbreak months for January in Bangkok would be 6/12 = 0.5. Figure 3 presents maps illustrating the proportion of HFMD outbreaks for each month over the 12-year study period. Conversely, a low proportion of high-risk outbreak months was observed in April and May. In Northern Thailand, the highest proportion of high-risk outbreak months was observed from June to November, spanning from the rainy season to the dry (winter) season. In contrast, the northeast and central regions exhibited the highest proportion of high-risk outbreaks only from June to October. In southern Thailand, the high-risk proportion persists longer, from June to February, due to this region experiencing a rainy season almost year-round.

### 3.2. Model Evaluation

**Figure 4:**
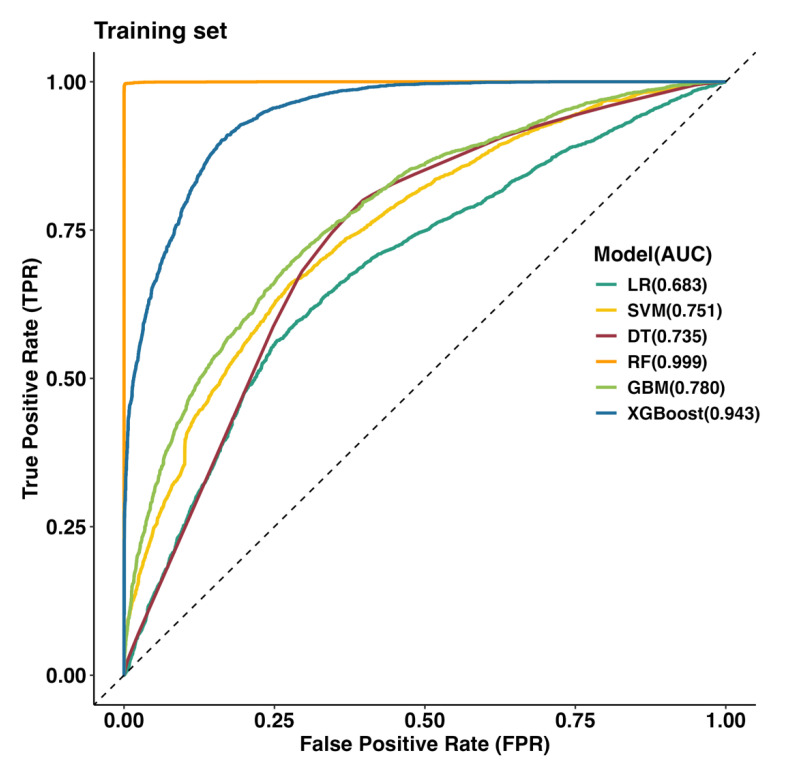
ROC curves predicting HFMD outbreaks for the training set (2011–2018).

Six models were evaluated using the AUC and five additional metrics. As shown in Table 3, the RF and XGBoost models demonstrated the best performance on the training dataset, with AUC scores exceeding 0.8 (0.999 and 0.943, respectively). These two models were selected to predict unseen data for the years 2019–2022. Figure 4 illustrates the Receiver Operating Characteristic (ROC) curves of the six models from 2011–2018. The RF model showed the highest performance on the training set, followed by the XGBoost model.

**Figure 5:**
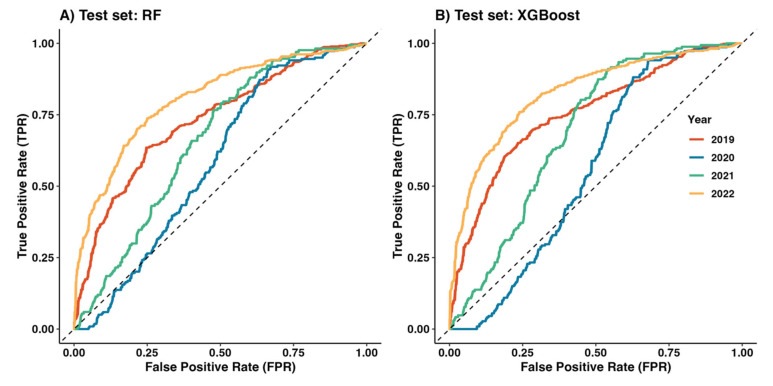
ROC curves predicting high-risk HFMD outbreaks for the test set (2019–2022) from (**A**) RF and (**B**) XGBoost models.

To account for the impact of the COVID-19 outbreak, testing was conducted separately for each year. The results indicated that the XGBoost model achieved the best performance (Table 4). Figure 5 presents the ROC curve for these predictions. In 2019, both the RF and XGBoost models demonstrated strong performance, achieving AUC scores of 0.730 and 0.786, respectively. However, during the COVID-19 pandemic in 2020, the predictive performance of both models declined, with AUC scores dropping to 0.583 for RF and 0.537 for XGBoost. Although the AUC scores improved in 2021, the models exhibited lower precision, suggesting that they predicted more positive high-risk outbreaks than actual. After the pandemic, in 2022, the models’ performance improved significantly, with AUCs of 0.806 for RF and 0.836 for XGBoost, indicating robust predictive capabilities.

**Figure 6:**
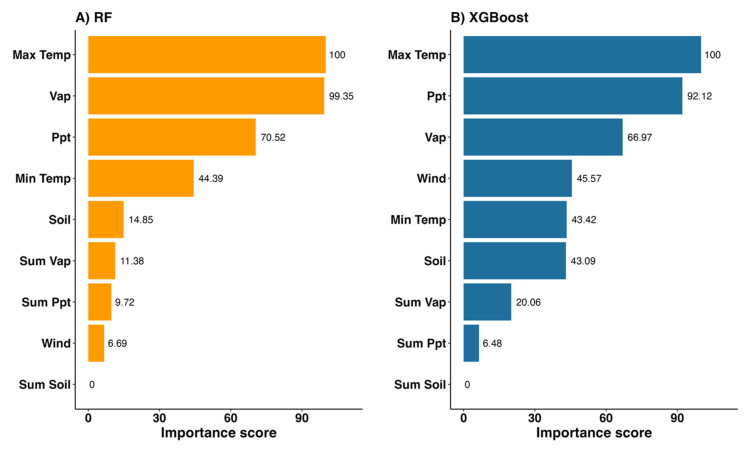
Variable importance rankings for (**A**) RF and (**B**) XGBoost models.

Using the RF and XGBoost models, the three most important variables were identified: average maximum temperature (Max Temp), average precipitation (Ppt), and average vapor pressure (Vap), as illustrated in Figure 6. Max Temp was the most important variable for predictions in both models, serving as the baseline for relative importance scores. Vap had relative importance scores of 99.35% and 66.97% in the RF and XGBoost models, respectively. Ppt had relative importance scores of 70.52% and 92.12% in the RF and XGBoost models, respectively. The remaining six features had relative importance scores below 50%, suggesting a lower contribution to the model predictions.

We examined the Pearson correlation among the meteorological variables (Figure S11). We found strong positive associations between average minimum temperature (Min Temp) and average vapor pressure (Vap), with correlation coefficients of *r* = 0.92 (*p* < 0.05). Max Temp was positively associated with Min Temp with *r* = 0.51 (*p* < 0.05). Additionally, Max Temp had a negative association with average soil moisture (Soil). Notably, the average and the summation of the precipitation and soil moisture exhibited a positive correlation, while Vap and Sum Vap showed a negative correlation.

### 3.3. Impact of the COVID-19 Pandemic on HFMD Outbreaks

**Figure 7:**
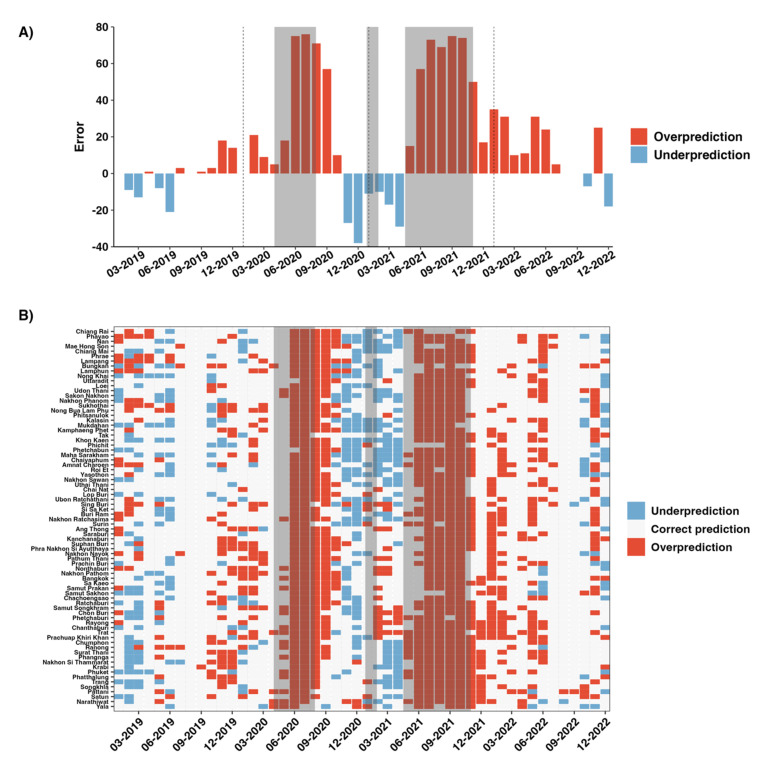
The error of predicted HFMD outbreaks across 77 provinces (**A**) and the heatmap of predicted HFMD outbreak months (**B**). Positive errors represent overprediction (predicted high-risk outbreaks higher than actual), while negative errors represent underprediction (predicted high-risk outbreaks lower than actual). The grey highlighted area shows the duration of the lockdown control measure [44,45].

Our best-performing model, the XGBoost, was used to predict high-risk HFMD outbreaks from 2019 to 2022. We calculated the errors by subtracting the predicted values from the actual values to assess the model’s accuracy. Figure 7A presents the errors for high-risk outbreaks across provinces and months. For example, in January 2021, there were 31 actual high-risk outbreak months and 20 predicted high-risk outbreak months for 77 provinces; the error would be −11. Positive errors indicated overprediction, where the predicted proportions were higher than the actual values, while negative errors indicated underprediction, where the predicted proportions were lower than the actual values. In 2019 and 2022, the error exhibited no systematic patterns across provinces, suggesting that the model’s predictions were unbiased (Figure 7B). This analysis validated the robustness of the model in accurately predicting HFMD outbreak proportions. Notably, in 2020 and 2021, overpredictions were observed across the country, especially during the lockdown periods.

## 4. Discussion

The performance of six machine learning (ML) models—LR, SVM, DT, RF, GBM, and XGBoost—was compared for detecting high-risk HFMD outbreaks in Thailand. Previous studies have applied multiple ML methods to classify HFMD outbreaks [22] and assess severe risk levels [46]. However, the majority of these studies have been confined to China. Our study aimed to identify high-risk HFMD outbreak areas in Thailand using meteorological data as input features. Our findings revealed that the RF model outperformed the other models in detecting high-risk outbreak months from the training data. However, the RF model demonstrated a tendency to overfit, resulting in lower performance on unseen data compared to the XGBoost model. XGBoost, a powerful ensemble model, has been widely applied in outbreak detection and has shown outperformance over other ML models. For instance, it has been used to predict outbreaks of the West Nile virus in Europe [21], dengue transmission rates [47], and the Rift Valley fever in Kenya [48]. XGBoost has also been extensively employed in other classification tasks, such as pre-slaughter mortality prediction for meat-type ducks in Thailand [49], PM2.5 concentration prediction in Shanghai [50], and highly pathogenic avian influenza outbreaks in poultry farms in South Korea [51]. Thus, XGBoost showed to be a promising method for classification tasks. It also performs a great potential tool for classification tasks.

The spatial distribution of high-risk HFMD outbreak proportions was analyzed to examine seasonal trends and evaluate the model’s performance in this study. We observed variations in the seasonal distribution of HFMD incidence rates across regions in Thailand. In Northern Thailand, high proportions were observed almost year-round, with peaks occurring from June to September. This finding is consistent with a previous study conducted from 2003 to 2012, which demonstrated that the disease occurs year-round, with peaks during the rainy and cold seasons [14]. In the central and southern regions, clearer seasonal trends were observed, with higher proportions occurring between June and September. This aligns with a prior study that reported HFMD incidence in the central region predominantly during the rainy season [13], further supporting these findings. The seasonal pattern observed in this study likely reflects the significant influence of meteorological factors on HFMD incidence.

In this study, we highlighted that the average maximum temperature was the most important factor in classifying high-risk outbreak areas. The average maximum temperature may play a crucial role in the absence of HFMD outbreaks in April (the summer season) when temperatures reach their peak. The high temperatures (39 °C) inhibit virus infectivity and activity, potentially limiting the spread of HFMD [52]. A previous study suggested that lower temperatures during the summer season might contribute to an increase in HFMD cases in Northern Thailand [14]. However, other studies in other temperate regions, such as Hong Kong [53], East China [54], and Japan [55], have contradicting results, with some finding a positive correlation between high temperature and HFMD outbreaks. Further investigation is needed to clarify the role of temperature in HFMD outbreaks in Thailand.

Average rainfall was identified as the second most important factor in the XGBoost model and the third in the RF model, consistent with previous findings in Northern Thailand [14] and Central Thailand [13]. High rainfall, which increases soil moisture, may contribute to the contamination of water sources with enteroviruses, potentially facilitating the persistence of the virus [56]. Our findings are consistent with previous studies. For instance, a study in Vietnam found that during the rainy season in 2012–2013, the number of HFMD cases increased significantly [12]. Similarly, HFMD admissions were associated with moderate rainfall in Hong Kong [57]. In this study, we also considered soil moisture, which is correlated with rainfall and could potentially contribute to HFMD outbreaks. However, it was not identified as a significant factor. An alternative explanation is that increased rainfall may reduce outdoor physical activity, leading to more indoor gatherings and consequently increasing the likelihood of contact with infectious individuals [58,59].

Vapor pressure was identified as the third most important factor in the XGBoost model (and the second most important factor in the RF model). Vapor pressure reflects the amount of moisture in the air and is closely correlated with rainfall. Increased rainfall raises vapor pressure, which may contribute to higher-risk outbreak areas during the rainy season. A study in Henan Province, China, revealed a significant association between the monthly mean water vapor pressure and HFMD cases [60]. Similarly, increased vapor pressure was shown to elevate the number of HFMD cases in Tokyo, Japan [61].

ML model successfully captured the high-risk HFMD outbreak areas in Thailand during the period prior to the COVID-19 pandemic (2011–2018). However, the model did not accurately predict the proportion of HFMD outbreak months in 2020 and 2021, as anticipated. During the COVID-19 pandemic in 2020–2021, the implementation of non-pharmaceutical interventions (NPIs) changes human behavior, affecting the transmission patterns of respiratory viruses, such as influenza [62], and vector-borne diseases, including dengue [63,64]. To control the spread of COVID-19 during the first wave in April 2020, the Thai government implemented several social measures, including the cancellation of national holidays to prevent mass gatherings and domestic travel, the closure of schools, and the restriction of access to public spaces, with exceptions made only for essential services [45]. Consequently, no high-risk HFMD outbreaks were observed during this period. However, as these measures were eased in July 2020, the proportion of outbreak months increased immediately, coinciding with the relaxation of COVID-19 restrictions and the reopening of schools.

The second wave of COVID-19 in January 2021 included a brief intervention that led to a rapid decrease in HFMD outbreaks, although they resumed afterward. Between May and October 2021, the response strategy shifted from a nationwide lockdown to provincial-level interventions based on daily COVID-19 case counts. Schools in most provinces, including Bangkok, were closed, while some provinces allowed schools to remain open. As a result, the proportion of HFMD outbreaks remained very low, even following the relaxation of restrictions. The proportion of HFMD outbreaks reached zero between February and April 2022, when no NPIs were in place. However, HFMD outbreaks sharply increased again in May 2022, a trend that our model was able to predict accurately.

In 2021, the actual high-risk HFMD outbreak months were significantly lower than the predicted outbreaks. This can likely be attributed to the NPI policies implemented in 2021, which were tailored provincially based on the daily COVID-19 case numbers in each province [45]. In high-risk provinces, schools and public areas were closed, while in some other provinces, they were allowed to open under specific controls. As a result, a small number of HFMD cases were observed in those provinces.

Even after the relaxation of NPIs, the overprediction of high-risk HFMD outbreaks was observed. The COVID-19 pandemic significantly altered human behaviors, which likely contributed to this sustained decrease in HFMD outbreaks [65]. People consistently adhered to preventive measures, including wearing face masks, maintaining social distancing, and regularly using alcohol-based hand sanitizers. Even kindergarten children wore face masks to school. As a result, HFMD cases did not increase immediately after the extended period of NPIs. However, in August 2022, HFMD outbreaks reemerged, recording the highest number of cases in the 12-year study period. This re-emerge may have been influenced by external factors such as climate change, the subtypes of the HFMD virus, and other variables, all of which warrant further investigation. A decrease in immunity levels against non-polio enteroviruses (NPEVs) may also be a contributing factor.

Although the XGBoost model in this study provides the best predictive performance using temperature, rainfall, soil moisture, vapor pressure, and wind speed features, there are several limitations. Due to data limitations across Thailand, we did not include air pollutants, which have been linked to HFMD infections [66], and relative humidity, which has been linked to HFMD in numerous previous studies [8,10]. The COVID-19 pandemic significantly changed human behavior and public health interventions, which may not reflect typical conditions and could limit the model’s predictive performance. The study did not account for long-term climate change effects. Additionally, it did not differentiate between HFMD virus subtypes, which could have different transmission dynamics due to the limited data. HFMD primarily affects children under the age of five; therefore, the opening and closing of schools, as well as the specific age groups affected, may significantly influence the seasonal pattern of HFMD. Lastly, the impact of specific NPIs and public health policies was not analyzed individually, which could also affect outbreak patterns.

## 5. Conclusions

This study developed a machine learning framework to predict high-risk HFMD outbreak areas in Thailand from 2011 to 2022 using meteorological features. Six ML models were evaluated, with XGBoost demonstrating the best overall performance in predicting high-risk outbreaks from unseen data. Our findings revealed the seasonal distribution of high-risk HFMD outbreak months across different regions. The average maximum temperature, average rainfall, and average vapor pressure were identified as important factors influencing predictions. The results highlighted regional differences in HFMD incidence patterns. The model effectively captured high-risk outbreaks before the COVID-19 pandemic but overprediction during 2020 and 2021, likely due to the control measures implemented at that time. Our study demonstrates the potential of ML models, particularly XGBoost, to enhance high-risk outbreak predictions using meteorological data. Future research may explore including more variables, such as air pollution and HFMD virus subtypes, to improve model performance. Integrating a spatial modeling framework could offer deeper insights into the geographic patterns of outbreaks and their relationships with environmental and social factors. Furthermore, evaluating the model’s performance at the provincial level could provide valuable insights into regional variations and enhance its adaptability for localized outbreak prediction and public health decision-making.

## Figures and Tables

**Table 1:** Descriptions of the meteorological variables.

Parameters	Descriptions
Min Temp	Average minimum temperature (°C)
Max Temp	Average maximum temperature (°C)
Ppt	Average precipitation (mm)
Soil	Average soil moisture (mm)
Vap	Average vapor pressure (kPa)
Wind	Average wind speed (m/s)
Sum Ppt	Sum of precipitation (mm)
Sum Soil	Sum of soil moisture (mm)
Sum Vap	Sum of vapor pressure (kPa)

**Table 2:** Descriptive analysis of the meteorological and HFMD incidence rate in Thailand during 2011–2022.

Variables	Mean	SD	Min	P25	Median	P75	Max
Incidence rate (per 100,000 people)	6.82	10.52	0	1.22	3.48	7.97	179.78
Min Temp (°C)	22.34	2.68	10.51	21.44	23.15	24.07	27.31
Max Temp (°C)	32.16	1.87	24.13	30.96	31.95	33.18	40.00
Ppt (mm)	129.52	121.01	0	23.77	106.88	197.75	981.67
Soil (mm)	161.42	93.68	7.98	77.24	151.33	235.29	409.67
Vap (kPa)	2.66	0.39	1.43	2.45	2.79	2.96	3.35
Wind (m/s)	1.36	0.49	0	1.04	1.32	1.64	4.02

**Table 3:** The performance metrics for the training sets (2012–2018).

Model	AUC	Accuracy	Precision	Recall	Specificity	F1
LR	0.683	0.629	0.604	0.412	0.794	0.490
SVM	0.751	0.685	0.676	0.523	0.809	0.751
DT	0.735	0.715	0.698	0.603	0.800	0.735
RF	0.999	0.995	0.997	0.993	0.997	0.995
GBM	0.780	0.712	0.695	0.596	0.800	0.780
XGBoost	0.943	0.870	0.906	0.782	0.938	0.839

**Table 4:** The performance metrics for the test sets (2019–2022).

Year	Model	AUC	Accuracy	Precision	Recall	Specificity	F1
2019	RF	0.730	0.680	0.746	0.702	0.646	0.620
2019	XGBoost	0.786	0.722	0.771	0.759	0.668	0.660
2020	RF	0.583	0.449	0.209	0.475	0.441	0.550
2020	XGBoost	0.537	0.485	0.260	0.635	0.438	0.565
2021	RF	0.660	0.389	0.117	0.365	0.394	0.513
2021	XGBoost	0.768	0.318	0.081	0.269	0.329	0.441
2022	RF	0.806	0.710	0.811	0.616	0.825	0.700
2022	XGBoost	0.836	0.669	0.888	0.455	0.930	0.602

## Data Availability

The data supporting the findings can be found in the main paper.

## Supplementary Materials

The following supporting information can be downloaded at https://www.mdpi.com/article/10.3390/tropicalmed10020048/s1, Table S1. The details of model training and hyperparameters for each model. Figure S1. A heatmap of Hand, Foot, and Mouth Disease (HFMD) incidence rates from 2011–2022. Figure S2. A heatmap of average of minimum temperature (°C) from 2011–2022. Figure S3. A heatmap of average of maximum temperature (°C) from 2011–2022. Figure S4. A heatmap of average of precipitation accumulation (mm) from 2011–2022. Figure S5. A heatmap of sum of precipitation accumulation (mm) from 2011–2022. Figure S6. A heatmap of average of soil moisture (mm) from 2011–2022. Figure S7. A heatmap of sum of soil moisture (mm) from 2011–2022. Figure S8. A heatmap of average of vapor pressure (kPa) from 2011–2022. Figure S9. A heatmap of sum of vapor pressure (kPa) from 2011–2022. Figure S10. A heatmap of average of wind speed (m/s) from 2011–2022. Figure S11. The Pearson correction coefficient between HFMD incidence rate (per 100,000 population) and meteorological features from 2011–2022.
