# Supplementary material for: Seasonal and Meteorological Drivers of Hand, Foot, and Mouth Disease Outbreaks Using Data-Driven Machine Learning Models
Source: Trop Med Infect Dis. 2025 Feb 6;10(2):48. doi: 10.3390/tropicalmed10020048 (PMC11860531; doi:10.3390/tropicalmed10020048)
Supplement: Supplementary file 1 [file tropicalmed-10-00048-s001.zip › tropicalmed-3448698-supplementary.pdf]

Supplementary Materials

Table S1. The details of model training and hyperparameters for each model.

Model	Method	Hyperparameters	Method details
**LR**	glm		
**SVM**	svmRadial	TuneLength =10,<br>line search	radial basis function kernel
**DT**	rpart	expand.grid(cp = seq(0.001, 0.1, by = 0.001))	Simple tree-based method
**RF**	ranger	expand.grid(<br>mtry = c(1:9),<br>splitrule = c("gini"),<br>min.node.size = c(1, 5, 10))	Includes permutation-based feature importance
**GBM**	gbm	expand.grid(n.trees = c(100),<br>interaction.depth = c(1, 3, 5),<br>shrinkage = c(0.01, 0.1, 0.2))	
**XGBoost**	xgbTree	expand.grid(nrounds = c(100, 200, 300),<br>eta = c(0.01, 0.1, 0.2),<br>max_depth = c(3, 6, 9),<br>gamma = c(0, 1, 5),<br>colsample_bytree = c(0.6, 0.8, 1),<br>min_child_weight = c(1, 3, 5),<br>subsample = c(0.7, 1))	

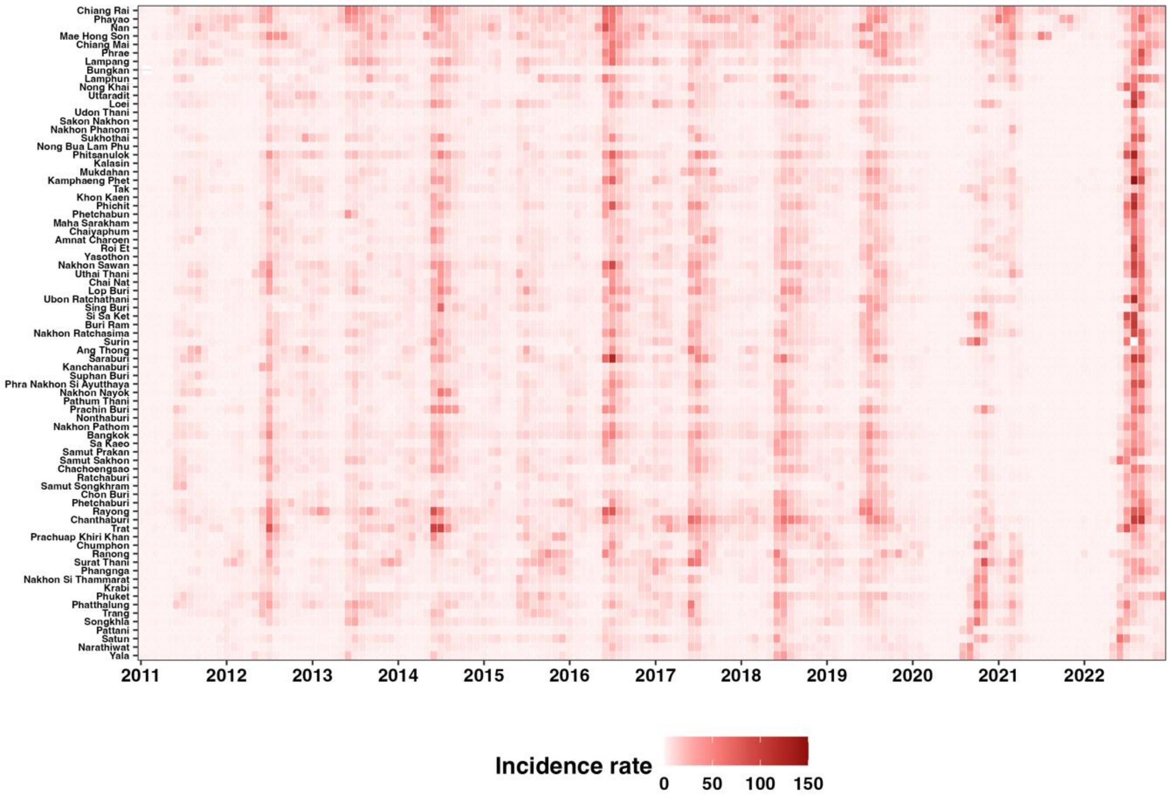

Figure S1. A heatmap of Hand, Foot, and Mouth Disease **(HFMD) incidence rates** from 2011-2022.

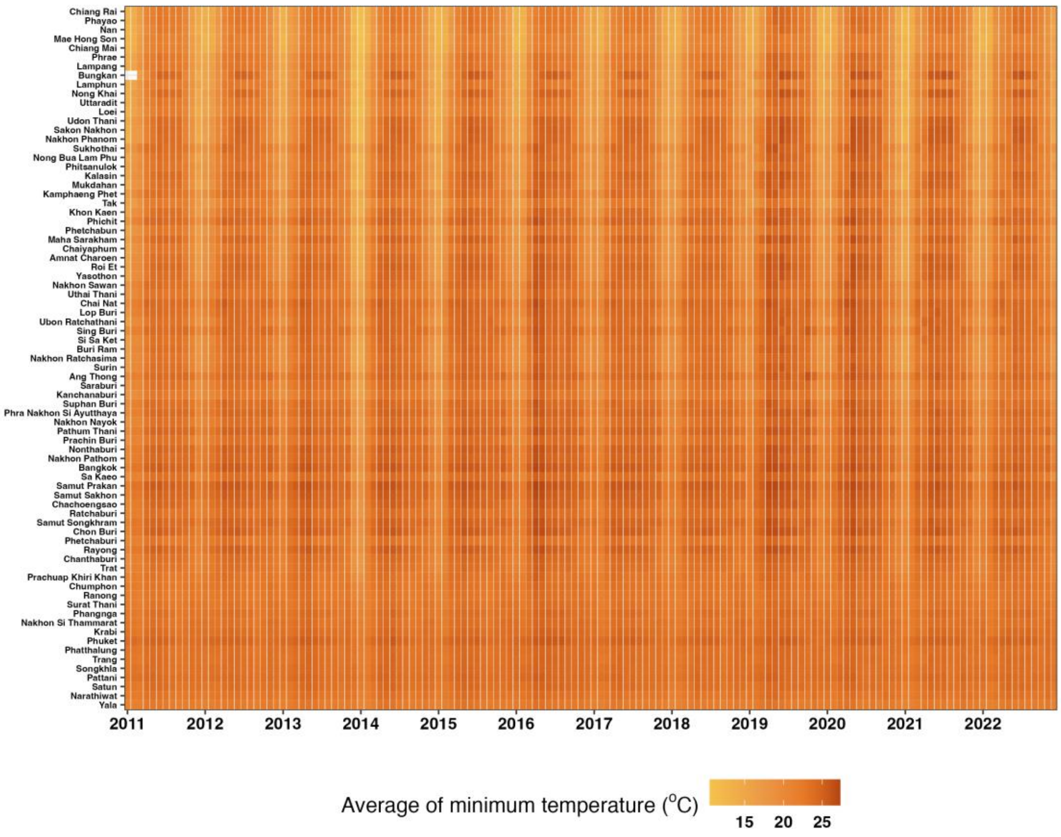

Figure S2. A heatmap of **average of minimum temperature** (°C) from 2011-2022.

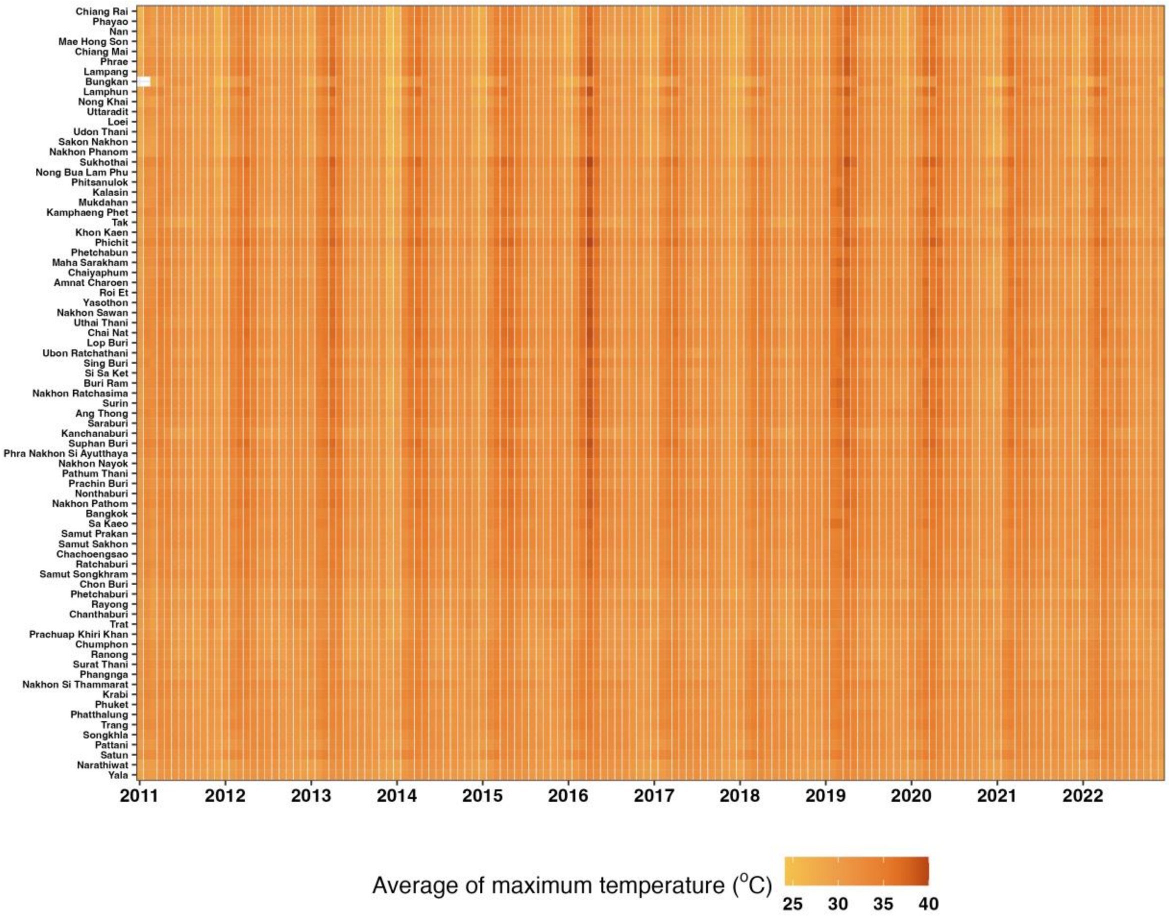

Figure S3. A heatmap of **average of maximum temperature (ºC)** from 2011-2022.

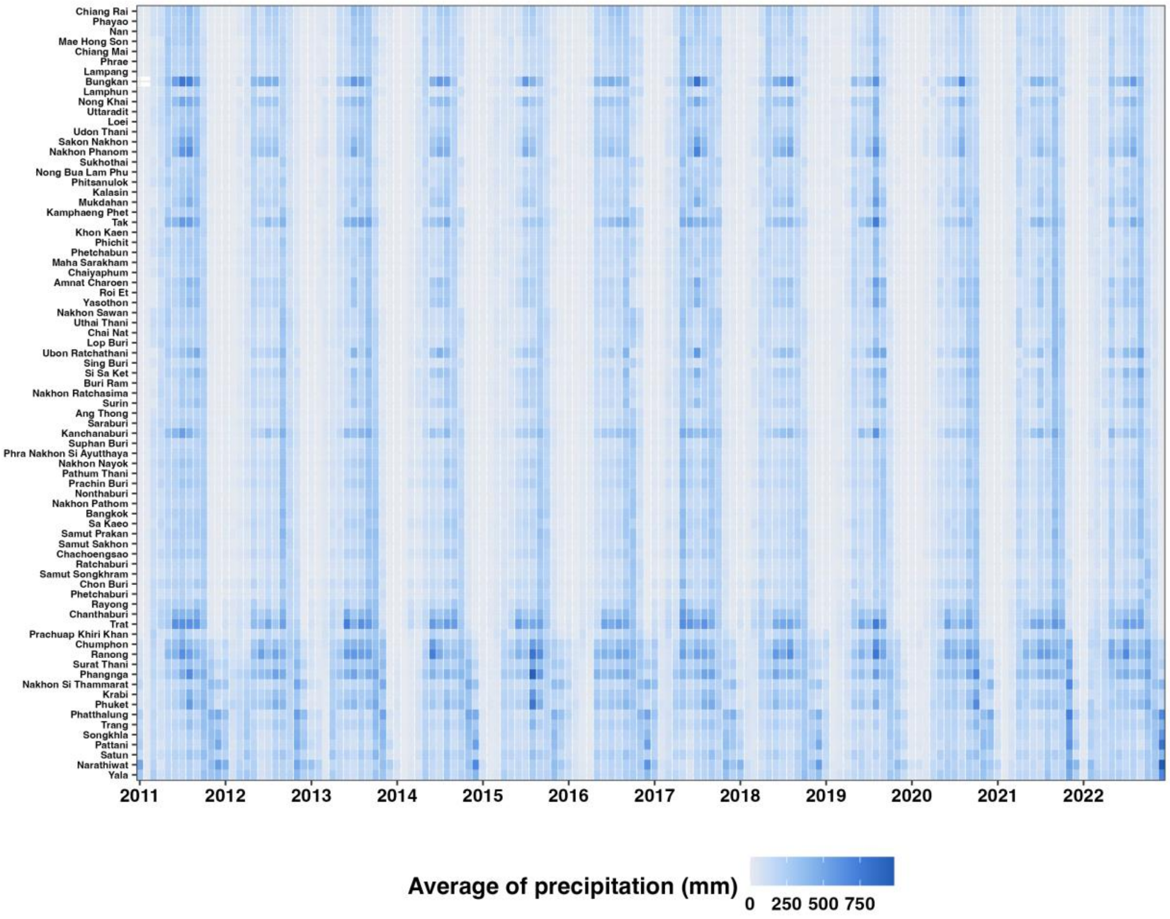

Figure S4. A heatmap of **average of precipitation accumulation (mm)** from 2011-2022.

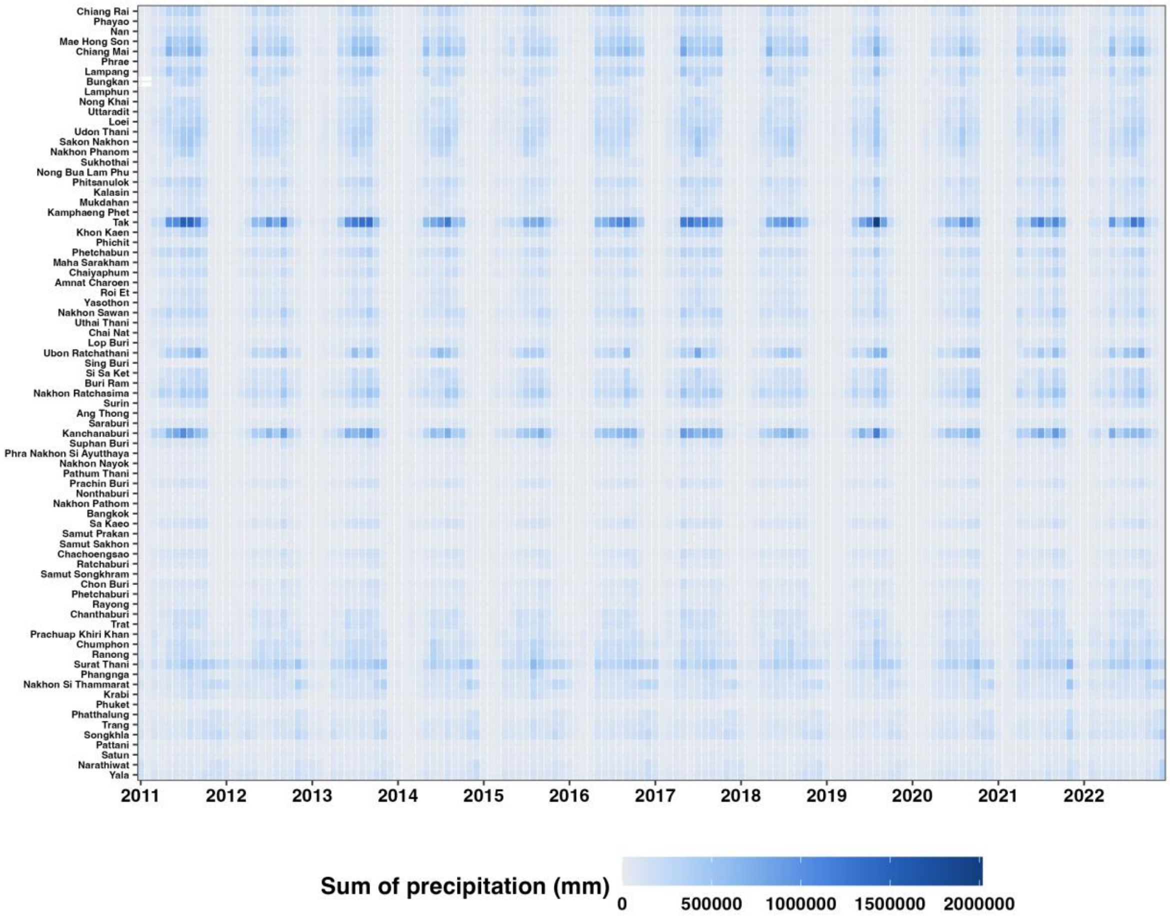

Figure S5. A heatmap of **sum of precipitation accumulation (mm)** from 2011-2022.

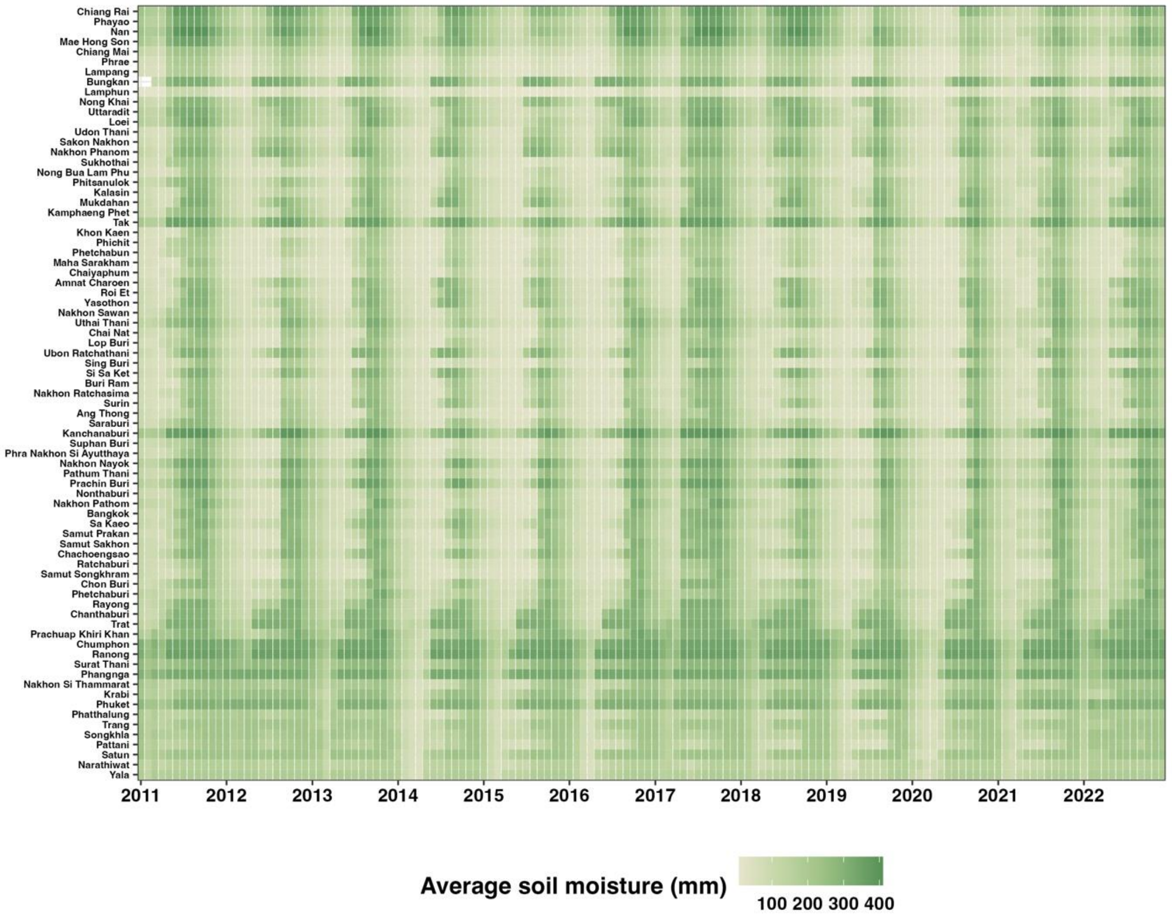

Figure S6. A heatmap of **average of soil moisture (mm)** from 2011-2022.

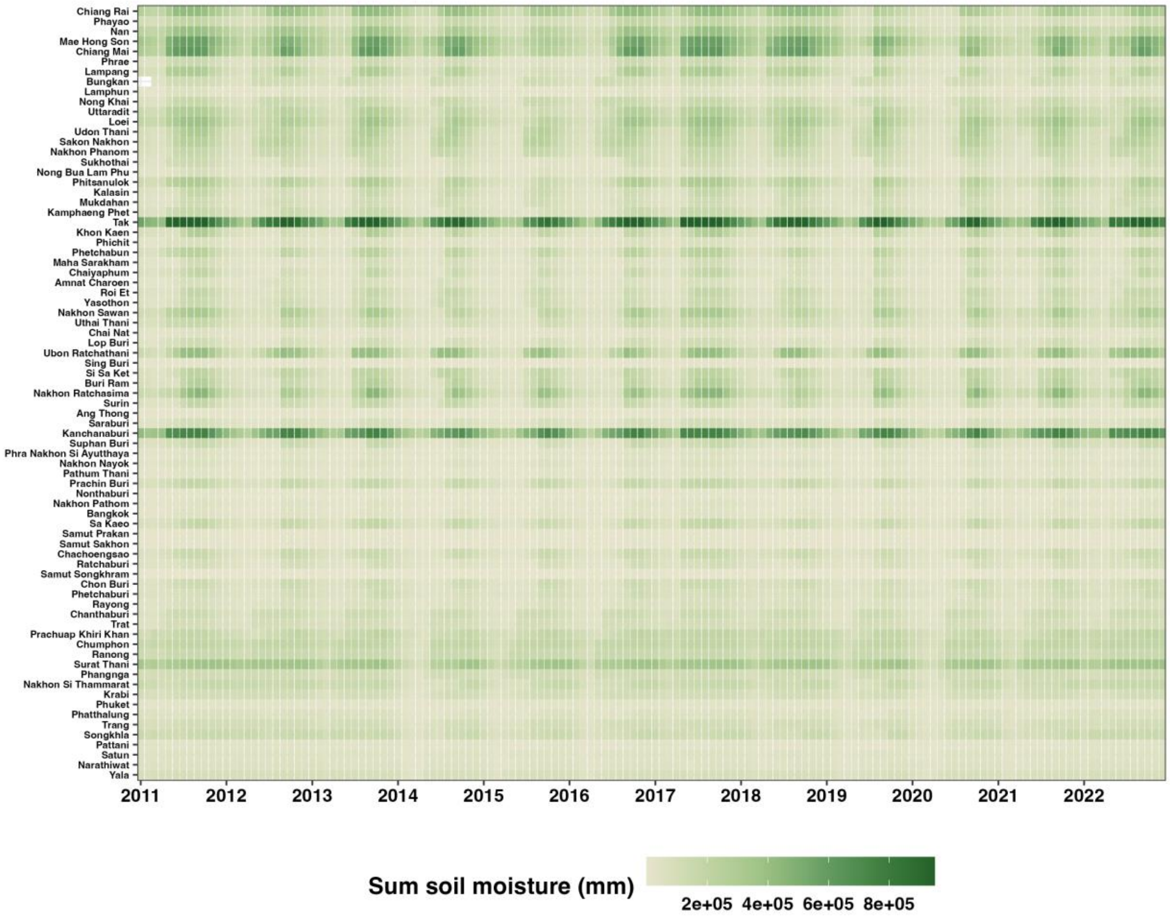

Figure S7. A heatmap of **sum of soil moisture (mm)** from 2011-2022.

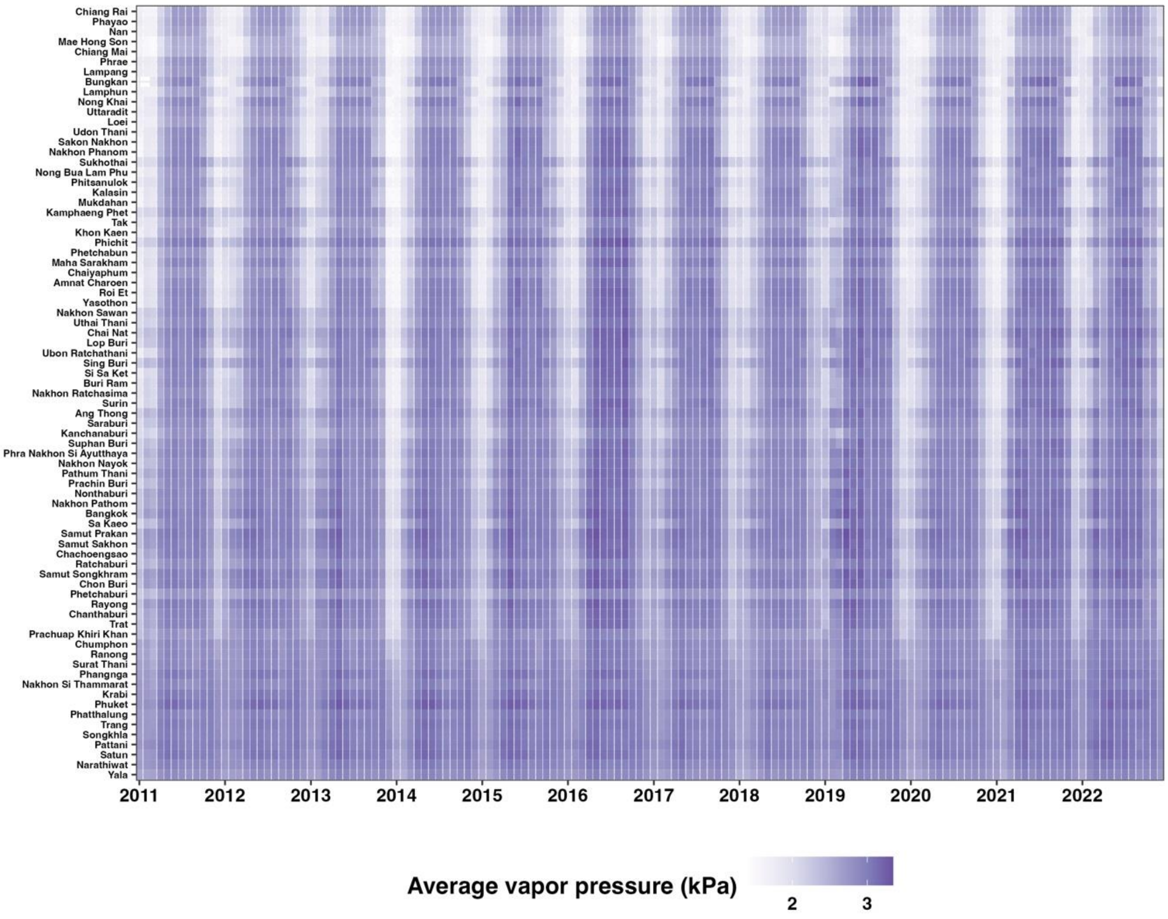

Figure S8. A heatmap of **average of vapor pressure (kPa)** from 2011-2022.

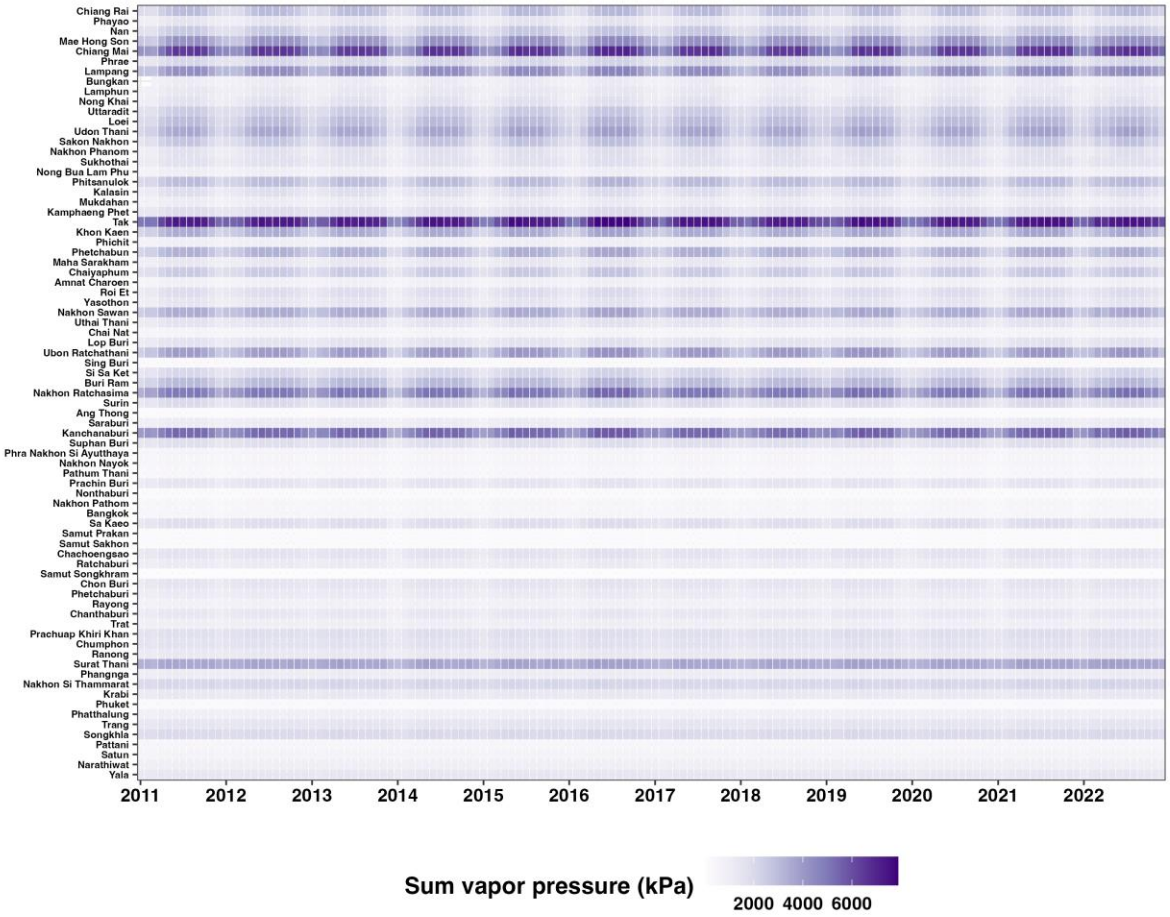

Figure S9. A heatmap of **sum of vapor pressure (kPa)** from 2011-2022.

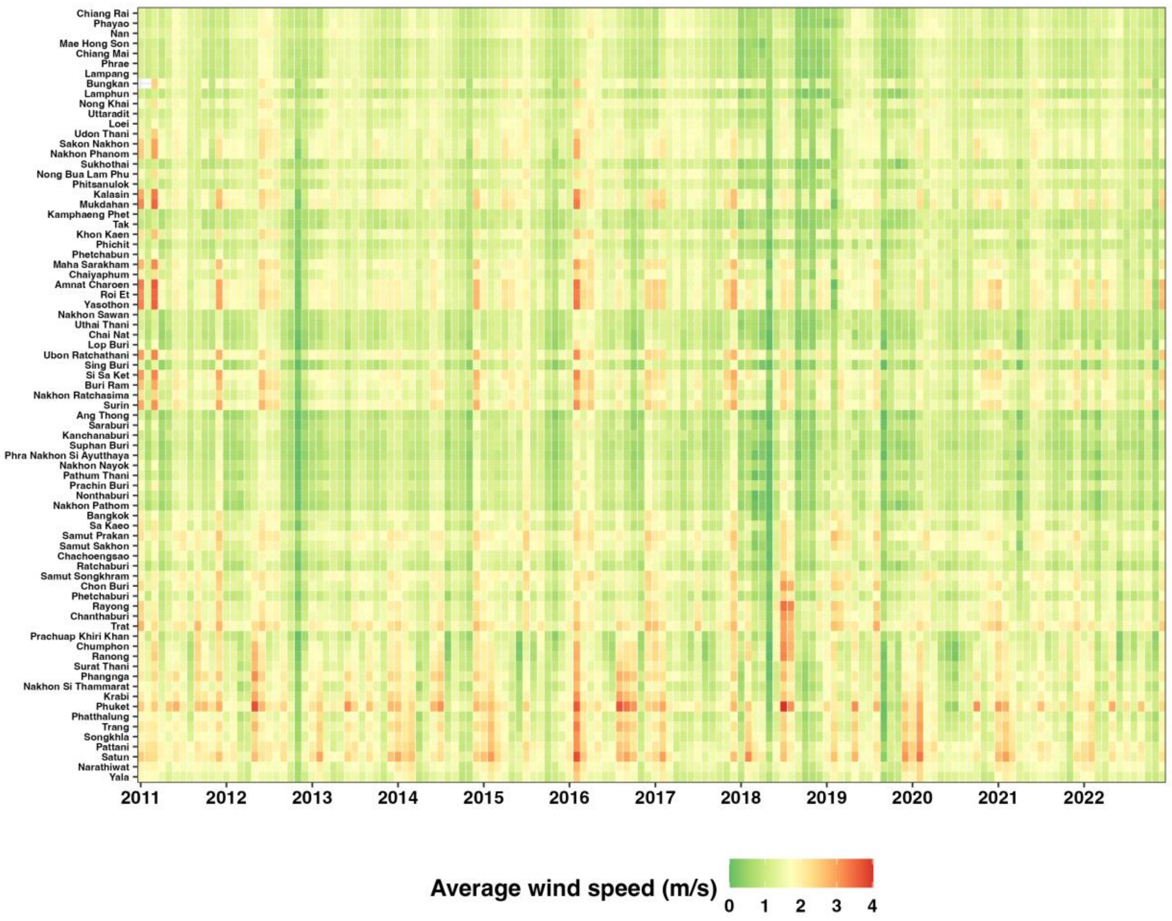

Figure S10. A heatmap of **average of wind speed (m/s)** from 2011-2022.

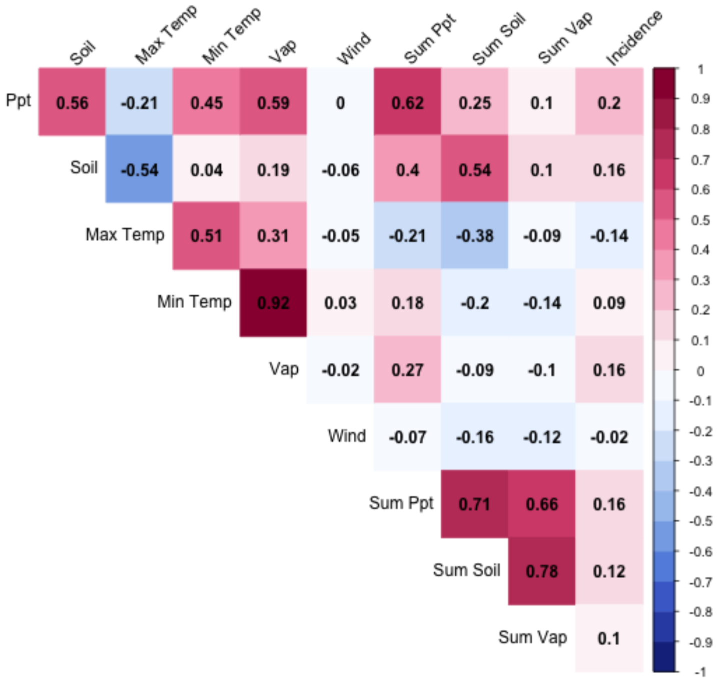

Figure S11. The Pearson correction coefficient between HFMD incidence rate (per 100,000 population) and meteorological features from 2011-2022.
